# Efficacy of omalizumab in children, adolescents, and adults with severe allergic asthma: a systematic review, meta-analysis, and call for new trials using current guidelines for assessment of severe asthma

**DOI:** 10.1186/s13223-020-00442-0

**Published:** 2020-06-18

**Authors:** Daniel P. Henriksen, Uffe Bodtger, Kirsten Sidenius, Niels Maltbaek, Lars Pedersen, Hanne Madsen, Ehm A. Andersson, Ole Norgaard, Louise K. Madsen, Bo L. Chawes

**Affiliations:** 1 Department of Clinical Biochemistry and Pharmacology, Odense University Hospital, Odense, Denmark; 2 Clinical Pharmacology and Pharmacy, Department of Public Health, University of Southern Denmark, Odense, Denmark; 3 Department of Respiratory Medicine, Næstved Hospital, Næstved, Denmark; 4 Allergi og Lungeklinikken Helsingør, Helsingør, Denmark; 5 Department of Medicine, Zealand University Hospital, Roskilde, Denmark; 6 Department of Respiratory Medicine, Bispebjerg Hospital, Copenhagen, Denmark; 7 Department of Respiratory Medicine, Odense University Hospital, Odense, Denmark; 8 The Danish Medicines Council Secretariat, Copenhagen, Denmark; 9 COPSAC, Copenhagen Prospective Studies on Asthma in Childhood, Herlev and Gentofte University Hospital, University of Copenhagen, Ledreborg Allé 34, Gentofte, 2820 Copenhagen, Denmark

**Keywords:** Severe asthma, Anti-IgE, Omalizumab, Systematic review

## Abstract

**Background:**

Omalizumab is approved for treating severe allergic asthma from age 6, but the definition of severe asthma including a systematic assessment to rule out difficult-to-treat asthma has changed since the drug was approved in 2003.

**Methods:**

We conducted a systematic review and meta-analysis of two critical (exacerbation rate, oral corticosteroid (OCS) treatment) and eight important clinical outcomes in children, adolescents and adults, and specifically searched papers for systematic assessment of severe asthma.

**Results:**

Adults: seven studies (n = 2159) ascertaining exacerbation rate showing a 37% (95% CI 21–50) reduction in favor of omalizumab, larger than the pre-specified minimal clinically important difference (MCID) of 25%. Only one open-label study (n = 82) was identified assessing the percentage of patients experiencing reduction of OCS-maintenance treatment showing a significantly greater decrease in the omalizumab group (− 45% vs. + 18.3%, p = 0.002). Children and adolescents: four studies (n = 1551) reported data on exacerbations (no meta-analysis conducted), showed overall improvements in exacerbation rate and some passed MCID. No OCS studies were identified. No included studies provided systematic assessment of severe asthma according to current guidelines.

**Conclusions:**

Omalizumab provides clinically relevant improvements in exacerbation rate among children, adolescents, and adults and in OCS-reduction among adults. New studies incorporating a guideline-approached definition of severe asthma are warranted.

## Background

Asthma is estimated to affect as many as 300 million people worldwide of whom most debuted in early childhood [1]. Many children, adolescents, and adults with asthma have poorly controlled disease and experience bothersome symptoms, frequent and severe exacerbations, reduced lung function, and airway hyperresponsiveness [2–5]. After systematic assessment to optimize asthma care [4, 5], including assessment of triggers, comorbidities and obstacles to asthma control, approximately 5–15% of the asthmatics remain to have severe asthma [6], which is associated with an increased morbidity and mortality and possess a significant socioeconomic burden [7].

Asthma is a heterogeneous syndrome consisting of several immunological subtypes [2, 4, 5] with one of the most well described being the allergic phenotype, which involves release of Th2 cytokines and production of immunoglobulin E (IgE) antibodies. Omalizumab is a recombinant DNA-derived humanised monoclonal antibody that selectively binds to human IgE [8], and is approved for severe allergic asthma from age 6 [8]. A previous Cochrane review from 2014 pooled paediatric and adult data and found favourable effects of omalizumab on the risk of exacerbations and reduction of ICS in severe allergic asthmatics [9]. However, there is increasing understanding of the pivotal role of multi-dimensional, systematic assessment of patients presenting with uncontrolled asthma to correctly phenotype the patient as either difficult-to-treat or truly severe asthma [2, 4, 5]. In fact, the majority of patients with uncontrolled asthma do not have severe asthma [2, 10] and according to the Nordic consensus statement on the systematic assessment and management of possible severe asthma in adults biological treatment including omalizumab is not recommended unless severe asthma has been diagnosed after systematic assessment [11]. Despite this, no previous review has included pre-omalizumab workup as part of the assessment of level of evidence.

Therefore, the Danish Medicines Council initiated a systematic review and meta-analysis on the efficacy and adverse events of omalizumab in both the paediatric and adult population aiming to assess the evidence of clinical effects for treating severe allergic asthma, including pre-omalizumab workup as part of the assessment of level of evidence.

The aim of the study was to assess the efficacy of omalizumab treatment in children, adolescents, and adults with severe allergic asthma.

## Methods

The Danish Medical Societies appointed an Expert Committee including experts in adult and paediatric asthma, clinical pharmacology, clinical pharmacy and patient representatives. The Expert Committee developed a protocol with predefined clinical questions structured as PICO (Patient, Intervention, Comparator, Outcome) questions and with predefined minimal clinically important differences (MCID) between omalizumab and placebo [12] and members of the Danish Medicines Council’s Secretariat thereafter aided with the literature search, selection of articles, data-extraction, and analysis.

### Protocol and registration

This systematic review and meta-analysis adheres to the Preferred Reporting Items for Systematic Reviews and Meta-Analyses (PRISMA) [13]. The protocol was drafted before the literature search [14] and the protocol and final report have been published in Danish on the Danish Medicines Council’s website [15, 16].

### Eligibility criteria

The literature search included all studies of children (6-11 years), adolescents (12–18 years) and adults (≥ 18 years) with asthma treated with omalizumab, to answer two predefined PICO questions:

1. Which adults (≥ 18 years) with severe allergic asthma should be offered treatment with omalizumab?
2. Which children and adolescents (6–18 years) with severe allergic asthma should be offered treatment with omalizumab?

The PICO questions are explained in detail in Additional file 1: Appendix.

Only studies using the approved dosing as subcutaneous administration were included for further evaluation. Studies were evaluated if they were randomised, but lack of blinding was not considered an exclusion criterion. Severe allergic asthma was defined based on the ERS/ATS guidelines by frequent exacerbations (at least two per year) despite treatment with high-dose inhaled corticosteroids (ICS) plus a second controller, or the need for daily oral corticosteroid treatment (OCS), to prevent exacerbations and achieve proper asthma control or stay uncontrolled on this treatment in a patient with aeroallergen sensitization, where allergy is considered the main trigger [2, 17]. Appropriate dosing was defined as the dosing presented in the Summary of Product Characteristics [18]

### Information sources and search strategy

MEDLINE, Embase and relevant databases from the Cochrane Library (see Additional file 1: Appendix, Table 1 for detailed search strings with annotations) were searched to identify systematic reviews and randomised controlled trials (RCTs). All databases were searched in MEDLINE (Ovid) so that records not yet MEDLINE-indexed were identified. Furthermore, we searched the National Guidelines Clearinghouse, Guidelines International Network, National Institute for Health and Care Excellence, Cochrane Library Technology Assessments (HTA), and the Danish Society of Respiratory Medicine to identify clinical guidelines. The Expert Committee and market authorisation holders were also invited to contribute with relevant literature.

**Table 1:** List of outcome measures

Outcome measures	Importance	Measure unit	Minimal clinically important difference
Mortality	Critical^a^		
Exacerbation rate	Critical	1. Average reduction in the annual number of exacerbations	1. 25% (a minimum reduction of 0.5 exacerbations per year)
		2. Number of patients who experience 0 exacerbations annually	2. 10 percentage points
Oral corticosteroid-maintenance treatment	Critical	1. Average %-reduction in daily dose (maintenance-treatment)	1. 20% (at least 2.5 mg prednisolone equivalent dose)
		2. Percentage of patients who are discontinued oral corticosteroid-maintenance treatment	2. 5 percentage points
		3. Percentage of patients who experience ≥ 50% reduction of oral corticosteroid treatment	10 percentage points^b^
Lungfunction FEV_1_	Important	1. Average change in lung function	1. 200 ml
		2. Percentage of patients who experience an improvement of 200 ml or more	2. 15 percentage points
Asthma control	Important	Average change in asthma control. A prioritised list of scores ACQ 5 (Asthma Control Questionnaire) ACT (Asthma Control Test) Other similar questionnaires	ACQ: 0.5 ACT: 3
Quality of life (QoL)	Important	Average change in QoL. A prioritised list of scores Asthma Quality of Life Questionnaire (AQLQ) Other questionnaires	AQLQ: 0.5
Serious adverse events (SAEs)	Important	The added number of SAEs	5 percentage points for the added number of SAEs
		Specific subgroups of SAEs, including anaphylaxis is assessed if they are distributed uniformly between the groups	No minimal clinically important difference is reported
Drop-out rate	Important	The percentage of patients who dropped out when the study was completed (difference between intention to treat population and difference between “intention to treat”-population and patients who completed the study)	10 percentage points
Sick leave	Important	Average number of sick leave days per year	5 days/year

For each outcome measure, the importance is indicated, and for critical and important outcome measures the minimal clinically important difference is reported

^a^Mortality is always considered to be a critical effect goal, albeit not an effective efficacy measure in the assessment of biological drugs in severe asthma. Asthma-related death occurs rarely, and it is therefore not estimated that outcome measure will provide any relevant information. In relation to safety, it is included in outcome measure: serious adverse events (SAEs). Mortality will therefore not act as a separate outcome measure in the assessment of the therapy

^b^The Expert Committee defined this outcome measure after the protocol was approved as data could not be extracted for the average OCS reduction

An information specialist from the Secretariat developed the search strategy based on input from the Expert Committee. The searches were conducted on 15 June 2017. Initially, the search strategy was designed also to include identification of records mentioning mepolizumab and/or reslizumab, but only records mentioning omalizumab were considered relevant for this review.

At least two members of the Expert Committee and the Secretariat manually screening the reference lists of the identified studies. References not already identified in the initial search were screened by full text reading.

An additional screening of PubMed for new omalizumab trials published from 15 June 2017 until submission of the manuscript did not reveal any new relevant studies.

### Study selection, data collection process and data items

Two persons from the Secretariat independently screened the identified guidelines and title and abstracts from systematic reviews and RCTs to evaluate whether they were relevant to answer the PICO questions. Thereafter, selected systematic reviews and RCTs were screened on full-text level by one from the Secretariat and at least one Expert Committee member, resolving any disagreements by consensus-based discussion. The methodological quality of eligible studies was assessed using the Cochrane collaboration’s tool for assessing risk of bias [19].

### Summary measures and synthesis of results

Intention-to-treat analyses with hazard ratios (HR), rate ratios (RR), odds ratios (OR), or relative risks for binary outcome measures were calculated. For continuous outcome measures, we used mean difference (MD) or standardised mean difference (SMD). No imputation on missing data was applied.

Meta-analyses with the inverse variance method with the assumption of random effects were applied. Statistical test for heterogeneity were performed (Cochran’s Q) and degree of heterogeneity was described with I^2^ statistic.

We chose to present results narratively, if it was not possible to conduct a meta-analysis due to lack of studies, heterogenous outcomes among others.

### Confidence in cumulative evidence

The GRADE approach was used to assess the quality of evidence [20]. The GRADE system assesses the quality of the evidence per effect measures across studies. Evidence is assessed in relation to five domains that have a bearing on trust power estimate: Risk of bias, Inconsistency, Inaccuracy (imprecision), Indirect evidence (indirectness), and Publication bias.

## Results

### Study selection

#### Guidelines and systematic reviews

A total of six clinical guidelines and nine systematic reviews were identified, but none of these had direct transferability to answer the PICO questions, and thus were not included for further analysis.

#### Primary literature

**Fig. 1:**
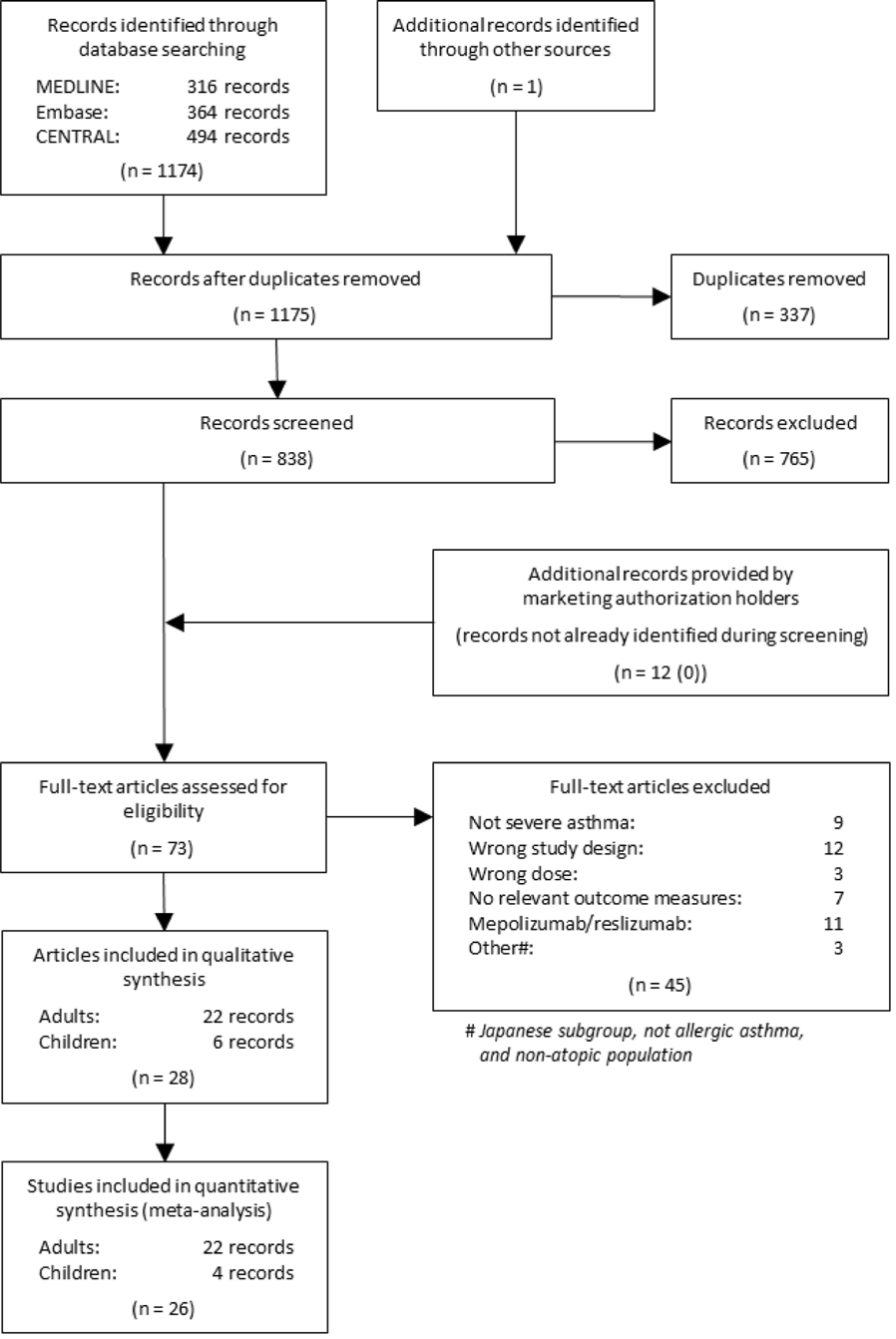
Flow chart of literature selection

The systematic search for RCTs yielded 1175 records. After removal of duplicates, screening and assessment for eligibility a total of 28 papers were included for further analysis (see Fig. 1).

Adults: We identified 22 published papers examining the effect of omalizumab in adults with severe asthma [21–42]. All studies had placebo or “standard of care” as comparators. One of the studies was a subgroup analysis of a RCT on patients with cat allergy [21].

Children and adolescents: Six papers [43–48] reported results from four RCTs [43–46] examining the effect of omalizumab in children and adolescents aged 6-18 years. Kulus et al. [47] was a subgroup analysis among children and adolescents with severe asthma from the main study by Lanier et al. [43], which also included moderate asthma cases, and Sorkness et al. [48] was a post hoc analysis of age, asthma severity, dosing regimen, and prespecified biomarkers from the study by Busse et al. [44].

We received 12 papers from the marketing authorisation holders, but they had already been identified through our database search. Nine of the studies were eligible for inclusion and three were excluded due to wrong study populations and design.

### Study characteristics

Both in the included studies of children, adolescents, and adults, study characteristics varied significantly between the included studies with regards to design, intensity of the standard of care asthma therapy, follow-up length, and number of previous exacerbations. Heterogeneity in results was examined in terms of difference in characteristics and design.

Among studies on adults, 12 studies were blinded RCTs (*n *= 4956), three studies were open-label clinical trials (*n *= 458), two studies were post hoc/sub-group analyses (*n *= 689), and four studies were extensions (*n *= 1508; two studies were extensions of the same RCT [*n *= 483]). Follow-up was between 16 and 52 weeks, 7 studies with only severe asthma, 6 studies with only moderate asthma. Among included studies on children and adolescents, four studies were blinded RCTs (*n *= 1551), and two were post hoc/sub-group analyses (*n *= 911). The studies varied in follow-up from 17 to 104 weeks, and one study only included patients with severe asthma. An overview of all included studies is presented in Additional file 1: Appendix.

Among studies evaluating the effect on adults, there was a risk of bias for all outcome measures except drop-out rate. Six studies [22, 24, 33, 37, 39, 40] had an unblinded design and thus high risk of bias. The two most methodically well-conducted studies had a low risk of bias in all domains [28, 44]. Among studies evaluating the effect on children and adolescents, the risk of bias was generally considered low.

### Synthesis of results

#### Exacerbations

##### Average reduction in the annual number of exacerbations

**Fig. 2:**
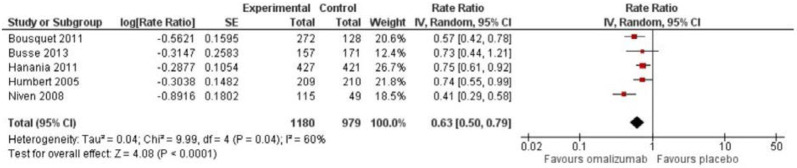
Rate ratio for annual exacerbation rate among adults

Adults: Seven RCTs reported the exacerbation rate, but only five studies had data, which could be included in the meta-analysis [24, 28, 31, 34, 37]. These five studies comprised a total of 2159 patients with a mean follow-up of 37 weeks (range 24–52 weeks). The rate ratio (RR) for the number of annual exacerbations showed a favourable effect in the omalizumab group compared to placebo: RR 0.63 (95% CI 0.50; 0.79), which can be translated into an absolute risk reduction of 37% (21; 50) (Fig. 2). Considering that the typical patient suffers at least two yearly exacerbations, the absolute reduction in exacerbations was 0.74 (0.42; 1.00), which was larger than the predefined MCID of 25%, i.e. 0.5 exacerbations per year, but the 95% CI overlapped this value. The quality of evidence was considered very low and heterogeneity was moderate (I^2^ = 60%, p = 0.04).

The study by Niven et al. added heterogeneity to the meta-analysis, and by excluding this study from the analysis, the RR was 0.70 (0.61; 0.81), but still significant in favour of omalizumab treatment.

Children and adolescents: Four studies were identified reporting on exacerbations, but it was not possible to combine the results into a meta-analysis because of differences in the presentation of the outcome measure [43–46] and therefore the results are presented narratively.

In the study by Lanier et al. including 627 children and adolescents with moderate to severe allergic asthma [43] exacerbations were defined as a worsening of symptoms requiring a doubling of baseline ICS-dose and/or rescue treatment with OCS ≥ 3 days. The risk of exacerbation was reduced with 31% (RR 0.69 [95% CI 0.53; 0.90]) after 24 weeks of treatment with omalizumab with concomitant stable treatment with ICS. For the subgroup of children and adolescents with severe asthma, assessed by Kulus et al. [47], the RR was 0.66 (0.44; 0.99), which was considered statistical significant and surpassed the MCID of 25%.

Sly et al. (N = 27) [46] found no difference in the frequency of moderate exacerbations, but in the treatment period of 5 months 1 out of 14 in the omalizumab group (7%) and 6 out of 13 in the placebo group (46%) experienced a severe exacerbation [49]. The uncertainties of these results are large, because of the low number of included children and adolescents. At a 2-year follow-up, no difference between the two groups was observed.

Teach et al. (N = 478) [45] observed a lower risk of exacerbations, defined as a worsening of asthma control requiring OCS or hospitalization, in the 90-day period beginning on the first day of each participant’s school year in the subgroup of children and adolescents treated according to GINA step 5: omalizumab versus placebo, 32.6% versus 15.1%, OR 0.37 [95% CI 0.17; 0.81]). The estimate surpasses the MCID, but with large uncertainty. No significant differences were observed among children and adolescents in GINA step 2–4 treatment.

In the study by Busse et al. [44] including 419 children and adolescents, the proportion of children and adolescents who experienced 1 or more exacerbations defined as a need for OCS, hospitalization, or both within the study period of 60 weeks was 30.3% in the omalizumab group compared to 48.8% in the placebo group. This surpassed the MCID of 10%-points, but the 95% CI overlapped this value.

##### Number of patients who experience 0 exacerbations annually

**Fig. 3:**
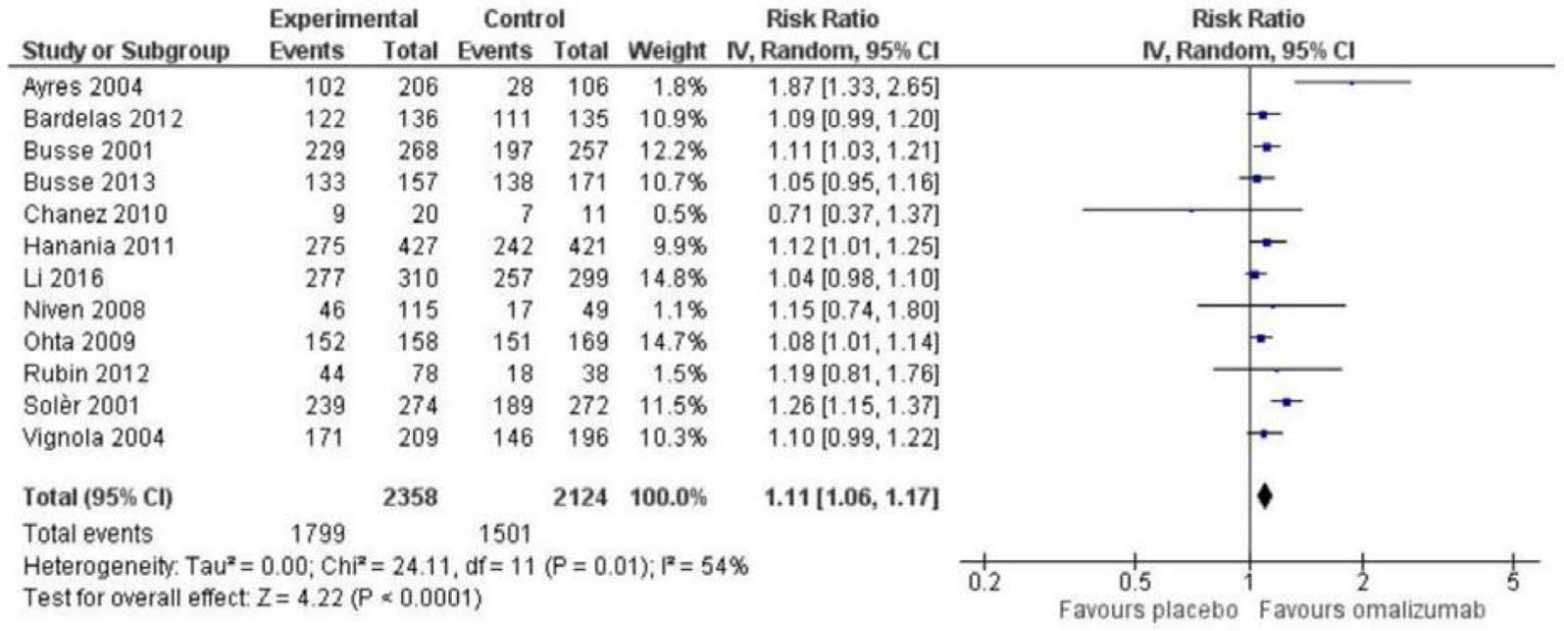
Risk ratio for percentage who experience 0 exacerbations among adults

Adults: In total, 12 RCTs comprising 4482 patients were included in the meta-analysis [22, 23, 27–29, 31, 36–39, 41, 42], showing a relative improvement of 1.11 (95% CI 1.06; 1.17) on the percentage of patients experiencing 0 exacerbations in favour of the omalizumab group (Fig. 3). The absolute difference was 8.2%-points (95% CI 5.2; 10.4) compared to placebo, which is less than the MCID of 10%-points. The results can be translated to 82 out of 1000 (95% CI 52; 104) persons achieving 0 exacerbations annually when treated with omalizumab over an average of 27 months, compared to placebo. The heterogeneity was moderate (I^2^ = 54%, p = 0.01) and quality of evidence low.

Subgroup analyses: A subgroup analysis of Busse et al. and Soler et al. (pooled data Massanari et al. 2009) showed that in adult patients with sensitization to cats, there was an effect of omalizumab on the risk of exacerbation: RR 0.50 (0.37; 0.67). A reduction of exacerbations was also observed in adult patients, who were mono-sensitised to cats [21, 27, 41]. No sub-group analyses in children and adolescents were identified.

#### Oral corticosteroid (OCS) use

A single study on adults with a 32 weeks follow-up was identified where OCS reduction was the outcome measure [40], which was a predefined subgroup analysis of Bousquet et al. [24]. The study was not blinded, examining omalizumab (N = 59) vs. “standard of care” (N = 23), where OCS consumption in each adult patient could be reduced after clinical assessment. We identified no studies regarding OCS treatment in children and adolescents and therefore the Expert Committee decided to include use of ICS therapy as a post hoc exploratory outcome measure in children and adolescents.

##### Median reduction and percentage of patients who experienced ≥ 50% reduction of OCS

Adults: In the omalizumab group, OCS-consumption was reduced by 45% (SD 50.22), while OCS-consumption in the control group was increased by 18.3% (85.13) after 32 weeks of treatment (p = 0.013). This estimate of the difference is above the MCID of 20% reduction, but it is not possible to assess the uncertainty as 95% CI was not provided. The quality of evidence was considered very low.

##### Percentage of patients who discontinued OCS

Adults: In the omalizumab group, 19 out of 59 patients discontinued OCS, compared to 3 out of 23 in the placebo-group, which accounted for a relative difference of 2.47 (95% CI 0.81; 7.55) in favour of omalizumab. This yielded a 19.2%-points difference (–2.5; 85.2%-points) in favour of omalizumab. The estimate was not statistical significant and is associated with a large degree of uncertainty. The quality of evidence was considered very low.

##### Inhalation corticosteroid (ICS) treatment

Children and adolescents: The post hoc outcome measure was defined as the proportion, who achieved to reduce ICS from high dose to moderate dose with a MCID of 15%-points.

For the subgroup with severe asthma from the study by Lanier et al. [43] no significant difference in the omalizumab group compared to the placebo group was observed. The reduction in fluticasone dose from baseline to 52 weeks including both the stabile and the steroid adjustment phase showed a reduction of 2.5% in the omalizumab group compared to 2.0% in the placebo group, i.e. below the MCID. Busse et al. [44] showed a statistically significant difference at study end, where the omalizumab versus placebo group were treated with 663 (SE 23.3) and 771 (23.5) µg budesonide equivalent/day, corresponding to a difference of -109 µg/day (95% CI − 172; − 45), p = 0.0012. Thus, both groups had a moderate dose at study end and the between group difference was not considered clinical relevant. The quality of evidence was considered very low.

#### Lung function

##### Adults

13 studies were identified with estimation of the effect of omalizumab on lung function [23, 24, 27, 28, 33–39, 41, 42]; five studies presented lung function with FEV1%- predicted [24, 27, 36, 37, 41]. Most studies did not present data sufficiently for a meta-analysis. Therefore, the results of the individual studies are presented narratively in the Additional file 1: Appendix. The Expert Committee concluded that an overall positive effect on FEV1 was observed in the omalizumab group, but not achieving clinical relevance. The quality of evidence was considered low.

##### Children and adolescents

In the three studies reporting lung function [44–46], no significant difference was observed between the omalizumab and placebo group. In the study by Busse et al. [44] the difference in FEV1%-predicted was 0.92 (95% CI − 0.81; 2.64) in favour of the omalizumab group, and deemed not statistically significant. Teach et al. [45] estimated lung function in FEV1%-predicted at the end of the study, adjusted for study site and dosage and found no statistical significant difference in either GINA treatment group. The quality of evidence was considered low. Sly et al. [46] presented no estimates of the effect on lung function, but concluded that no statistically significant or clinically relevant difference was observed.

#### Asthma control

##### Adults

**Fig. 4:**
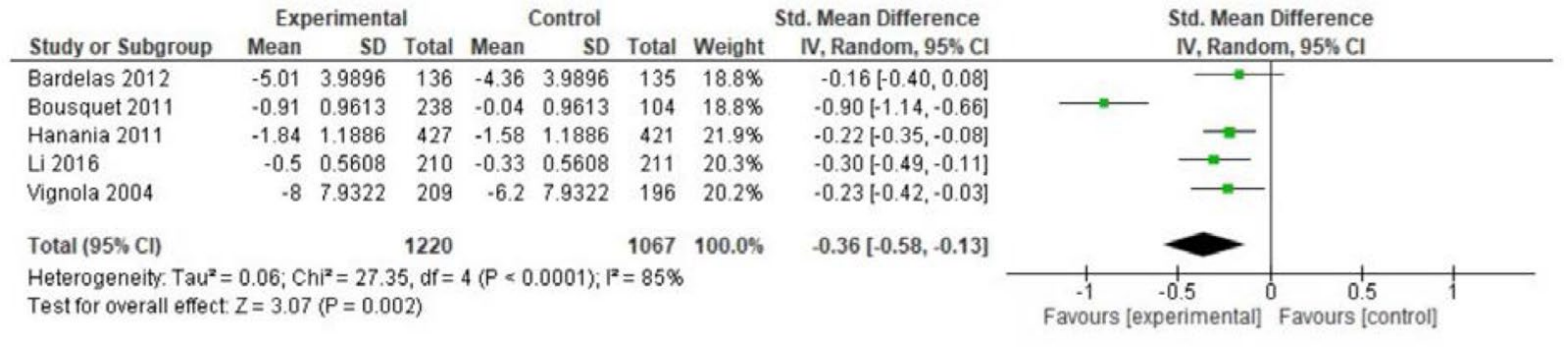
Mean difference asthma control (ACQ) among adults

Fourteen studies were identified with estimation of the effect of omalizumab on asthma control. The meta-analysis was performed based on five studies of 2287 patients [23, 24, 31, 36, 42] using ACQ and ACT and showed a SMD of − 0.36 points (95% CI − 0.58; − 0.13) in the omalizumab compared to placebo group. This was statistically significant, but below MCID of 0.5 points (Fig. 4). Serious heterogeneity was observed (I^2^ = 85%, p < 0.0001), where especially the study by Bousquet et al. 2011 contributed to this. When excluding that study from the meta-analysis, the SMD was − 0.23 ( − 0.32; − 0.14). The quality of evidence was considered very low. Results from the other nine studies on asthma control are described narratively in the Additional file 1: Appendix.

##### Children and adolescents

Two studies including 897 children reported the outcome asthma control. In Busse et al. [44] asthma control was assessed in subgroups of children 4-11 years and adolescents 12-20 years, measured by the C-ACT/ACT score, in the last month of follow-up. Among the youngest children (assessed by C-ACT) a difference between omalizumab and placebo after 48 weeks of treatment was reported as 0.78 points (95% CI 0.21; 1.35), which was statistically significant, but the MCID for C-ACT is not known. For the oldest group of children and adolescents, a difference of 0.19 points (0.42; 0.79) was observed, which was not statistically significant and below the MCID for ACT of 3 points. Teach et al. [45] found a similar effect on asthma control measured by ACT and C-ACT in the subgroups treated according to GINA 5 and GINA 2-4. However, only among adolescents ≥ 12 years treated according to GINA 5, a statistically significant difference was observed: 1.28 points (0.08; 2.48), but not surpassing the MCID. The quality of evidence was considered very low.

### Quality of life

#### Adults

Ten studies were identified using the Asthma Quality of Life Questionnaire (AQLQ), and four studies comprising 1852 patients were included in the meta-analysis (Additional file 1: Figure S1). A significant improvement among patients in the omalizumab group compared to the placebo group was observed with a MD of 0.58 points (95% CI 0.06; 1.11), which was above the MCID of 0.5. Severe heterogeneity was observed (I^2^ = 95%, p < 0.00001). The study by Rubin 2012 [39] contributed to the heterogeneity, and if removed, MD was 0.29 (0.19; 0.39). The quality of evidence was considered very low.

#### Children and adolescents

Only one study was identified, which showed no statistically significant difference in quality of life (QoL) after 24 weeks of omalizumab treatment and a stable treatment with ICS compared to placebo, measured with PAQLQ [43]. The quality of evidence was considered very low.

### Drop out rate

#### Adults

We only included data from the blinded RCTs constituting eleven studies of 4557 patients [23, 26, 28, 29, 31, 32, 34–36, 38, 42]. The most frequent reason for drop out was withdrawal of consent followed by adverse events. Other reasons included lost to follow-up, and administrative problems. The meta-analysis showed a larger drop out rate in the placebo compared to the omalizumab group with a relative risk reduction of 0.77 (95% CI 0.59; 1.01). Recalculated to absolute values, we found − 2.7%-point ( − 4.7; − 0.1) difference in drop out in the omalizumab compared to the placebo group (Additional file 1: Figure S2a), which was below the MCID of 10%. We found moderate heterogeneity (I^2^ = 59%, p = 0.06) and the quality of evidence was considered low.

#### Children and adolescents

We identified five studies in four papers [44–47] (the study by Teach et al. was divided in children and adolescents with GINA 5 [Teach 2015a] and children and adolescents in GINA 2–4 [Teach 2015b]), comprising 1056 children and adolescents. The most frequent reason for drop out among children and adolescents was withdrawal of consent followed by lost to follow-up. Other reasons included adverse events, but also anaphylaxis. No statistically significant difference in drop out rates was observed: RR 0.83 (95% CI 0.56; 1.22) (Additional file 1: Figure S2b). Heterogeneity was considered low (I^2^ = 0%, p = 0.87), and the quality of evidence was considered moderate.

### Serious adverse events (SAE)

#### Adults

We included 13 studies of 5108 patients [22, 24, 26, 28, 29, 31, 32, 34–36, 38, 39, 42], and found no significant difference in the proportion of SAE in the omalizumab group versus placebo, with a RR of 0.85 (95% CI 0.69; 1.04). This was recalculated to an absolute value of − 0.8%-points ( − 0.7; − 3.8) (Additional file 1: Figure S3a), which was not greater than the MCID of 5%-points. We found no heterogeneity (I^2^ = 0%, p = 0.77). The quality of evidence was considered low.

#### Children and adolescents

We identified three papers [44, 45, 47], comprising 1040 patients. We found a statistically significant lower risk of SAE in the omalizumab group compared to placebo: RR 0.40 (95% CI 0.24; 0.67), which was above the predefined MCID (Additional file 1: Figure S3b). Heterogeneity was considered low (I^2^ = 0%, p = 0.72) and the quality of evidence was considered moderate.

### Days off work/school

#### Adults

Three studies presented data on sick-leave [22, 23, 29], but none of those could be included in a meta-analysis. The Expert Committee concluded that the data on sick leave was insufficient and could not be included in the assessment of omalizumab.

#### Children and adolescents

Sick leave, in terms of “missed school days” during 48 weeks of treatment was evaluated by Busse et al. [44] showing a difference of -0.09 days (95% CI -0.18; -0.01), which was below the MCID of 5 days/year. “Missed school days” was assessed in percentage of the 90 treatment days in the study by Teach et al. [45]. Among the group of children and adolescents in GINA 5, 1.4% (SD 0.36) equivalent of 1.26 days, and 3.2% (6.71) equivalent of 2.88 days was observed in the omalizumab group and placebo group, respectively. The difference was 1.62 days ( − 3.83; − 1.36), which is difficult to assess whether it is above or below the MCID threshold. The quality of evidence was considered moderate.

### Systematic, multidimensional assessment of possible severe asthma

The “Methods” section of all included papers were carefully searched for descriptions of workup prior to entering the study, and/or workup after inclusion prior to treatment onset. No study of either adults or children and adolescents described any assessment for asthma triggers, comorbidity, asthma mimickers, medication adherence, or other parameters [4, 5] essential for distinguishing difficult-to-treat asthma from truly severe asthma.

## Discussion

### Summary of the evidence

The current evidence base for omalizumab treatment to prevent exacerbations is not directly transferable to “real world” clinical practice due to a general lack of systematic assessment of the included patients to distinguish between poorly controlled difficult-to-treat asthma and severe asthma [11]. Furthermore, several studies also included patients with only moderate disease, to whom omalizumab is not recommended in clinical practice in Europe [8]. Overall, the evidence quality for all outcomes including the critical outcomes exacerbation rate and OCS maintenance reduction was considered low or very low.

Although the omalizumab data available are increasing in the adult population, it is extremely limited in the adolescent population and even more limited in those < 12 years of age. In the era of new evolving biologicals to target specific inflammatory phenotypes of severe asthma [50] there is an imminent need for new trials with strict systematic assessments and better case definitions to determine the benefits of the drugs in the true severe asthma population, which are seen in the respiratory outpatient clinics. Of importance, since omalizumab was first approved by FDA in 2003 the definition of severe asthma has changed and now requires a systematic assessment to rule out difficult-to-treat asthma and does not include a lung function criterion anymore. Thisposes a significant difference in the study populations in older vs. newer trials and may account for some of the heterogeneity observed across the RCTs [51].

#### Adults

The evidence following the evaluation of the critical outcomes showed a significant and clinical relevant effect of omalizumab in reduction of exacerbation rates among adults, who in the studies are described as having severe allergic asthma. The absolute reduction in exacerbations was 37% and considering that the typical severe asthma patient suffers at least two yearly exacerbations, our finding would imply a reduction of at least 0.74 exacerbations per year. In addition, we observed a significant 8.2%-points reduction in the proportion of patients, who achieved 0 exacerbations, which was below the prespecified clinically relevant 10%-points reduction. The findings for omalizumab on exacerbations are important as exacerbations are associated with lower QoL for the affected patients, increased morbidity and mortality and also account for a major draw on health care resources due to doctor contacts, ED visits, hospitalizations, and drug prescriptions [52]. However, for both exacerbation outcomes in our meta-analysis the confidence limits overlapped with the MCID, and a risk of bias as well as heterogeneity was observed.

The effect of omalizumab on OCS maintenance therapy was only evaluated in a single study, which showed a clinically relevant effect on the reduction of daily OCS dose, which is of great importance to reduce the well-known systemic side effects of such treatment including development of osteoporosis, diabetes, fractures, and cataract [53]. However, this effect did not achieve statistical significance on the percentage of patients who were able to discontinue OCS treatment, and no statistical analysis was performed on the average reduction of OCS dose. Thus, the assessment of the outcome measure OCS is solely based on data from a single study of 82 patients and therefore great uncertainty about the estimates exists. Further, the study was not blinded, and a systematic regime for the withdrawal of OCS was not predefined, which poses a risk of bias. New, larger and blinded RCTs are needed to investigate the effect of omalizumab on this critical outcome.

None of the eight important outcome measures were associated with a clear clinically relevant effect of omalizumab compared to placebo. An improvement was observed in favour of omalizumab for lung function and asthma control, but the differences were below the MCIDs. The results from most studies on QoL do not indicate a clinically relevant effect, while results from some studies suggest a clinically relevant effect. There is insufficient data on sick leave, and this is therefore not included in the assessment. We considered the evidence indirect in relation to the patient population of interest and the underlying systematic assessment and standard treatment of the included patients were not directly transferable to a “real world” clinical setting. There is a risk of bias due to lack of blinding and ambiguity about randomization for all the important effects on proximity. There are inconsistency and inaccuracy in the results for asthma control and QoL. Evidence quality was low or very low for the important outcome measures.

#### Children and adolescents

In general, only very limited evidence regarding the efficacy and adverse effects of omalizumab exists in the paediatric and adolescent populations. The evidence following the evaluation of the critical outcomes in the included studies of children and adolescents showed a clinically relevant effect of omalizumab in reduction of exacerbations of 31% [43], and up to 44% in the sub-group of children and adolescents defined by the authors as having severe allergic asthma [47]. However, the confidence limits overlapped with the MCID of 25%, and data was limited to a single study of 627 children and adolescents, which increases the risk of bias and heterogeneity. This was also the case with the critical outcome of patients achieving 0 exacerbations, which was presented in a single study of 419 American inner-city children and adolescents, characterized by low socioeconomic conditions and a mixed ethnicity with many African Americans [44]. Thus, this study differed significantly from the Danish setting both with respect to demography, but also in regard to the asthma severity as one-fourth of all included children and adolescents were identified to have mild asthma (GINA 1–2) at inclusion. This is in opposition to the GINA treatment guidelines, which are followed in Denmark, where omalizumab is only offered to children and adolescents who after systematic assessment has severe allergic asthma treated according to GINA 4–5.

We did not identify any studies evaluating the effect of omalizumab on OCS maintenance reduction in children and adolescents, which is also rarely used in this population, but a post hoc analysis on ICS reduction showed no clinically relevant effect compared to placebo in the two studies investigating such outcome.

None of the important outcome measures were associated with a clear clinical relevant effect of omalizumab compared to placebo in the identified paediatric studies. Importantly, no effect of omalizumab was observed on lung function, which contrasts the adult studies, and underscores that omalizumab should be prescribed to children and adolescents with severe allergic asthma to reduce their exacerbation risk, but not to improve their lung function. Further, this is an example showing that evidence from adult studies cannot be extrapolated to the paediatric population. A statistically significant effect in the outcome measures asthma control and SAE was observed, but not deemed clinical relevant. No statistical significant effect was observed in the outcome measures QoL, drop out rate or number of missed school days.

### Future perspectives

There is a clear need for new RCTs using systematic assessment at enrolment to differentiate between difficult-to-treat asthma and severe asthma [54]. Inclusion of difficult-to-treat asthmatic patients in RCTs will presumably lead to bias towards the null; i.e. overlooking or underestimating drug effects, as adherence will improve in both treatment groups when patients participate in trials. Inclusion of mild to moderate asthma is not relevant from a clinical perspective as the drug is not approved in such population.

Furthermore, there is a general need for studies in the pediatric population to better assess the effect on exacerbations and reduction in ICS maintenance treatment. In adults, there is a need of studies assessing the effect on OCS treatment designed similar to newly published reports on e.g. anti-IL5 drugs [55].

So, the question remains: How can our guidelines recommend with confidence and evidence, if the data available for the first biological agent are limited for the parameters that we have labelled as being important in the treatment of asthma/severe asthma? Considering cost issues and what our needs are at present in the severe asthma field, where new biologicals are being developed continuously, it would be perhaps more cost-effective to focus on head-to-head studies with other biological agents rather than having another study of a single biological agent vs placebo. Newer trials should preferably compare placebo to both omalizumab and new anti-IgE drugs in the pipeline (such as ligelizumab, which may be more potent than omalizumab [50]), as well as focus on head-to-head trials in “real world” settings. This also implies addressing the “real world” problems such as the fact that some patients on biologics taper down or even stop taking their controller medication such as ICS, which may lead to a poorer clinical response to the biological agent; addressing the fact that some patients need switching from one biological to another, where efficacy of such regime needs to be investigated in clinical trials; and ultimately comparing treatment efficacy of biologicals in patients with severe asthma vs. difficult-to-treat asthma as the latter may also benefit from biologicals and could be a target group if the cost of biological treatment drops in the future.

## Conclusions

The level of evidence of omalizumab in severe, allergic asthma is low to very low as the asthma populations included are not diagnosed with severe asthma according to current definitions. However, omalizumab appears safe and is associated with a significant and clinically relevant reduction on exacerbation rate and OCS maintenance dose, but the effect on lung function, asthma control and QoL is uncertain. There is imminent need for studies on omalizumab in both adult and paediatric patients who have true, severe asthma.

## Supplementary information

**Additional file 1.** Appendix.

## Data Availability

Raw data are available upon request

## Abbreviations

OCS: Oral corticosteroid
MCID: Minimal clinically important differene
IgE: Immunoglobulin E
PICO: Patient, intervention, comparison, outcome
PRISMA: Preferred reporting items for systematic reviews and meta-analyses
ERS/ATS: European Respiratory Society/American Thoracic Society/American
ICS: Inhaled corticosteroids
RCT: Randomised controlled trials
HTA: Cochrane library technology assessments
HR: Hazard ratio
RR: Rate ratio
OR: Odds ratio
MD: mean difference
SMD: Standardised mean difference
GRADE: Grading of recommendations assessment, development and evaluation
