# Supplementary material for: Efficacy of omalizumab in children, adolescents, and adults with severe allergic asthma: a systematic review, meta-analysis, and call for new trials using current guidelines for assessment of severe asthma
Source: Allergy Asthma Clin Immunol. 2020 Jun 18;16:49. doi: 10.1186/s13223-020-00442-0 (PMC7302157; doi:10.1186/s13223-020-00442-0)

# Appendix

1. Which adults (≥18 years) with severe allergic asthma should be offered treatment with omalizumab?
   1. Population - Patients ≥ 18 years of age with severe, allergic asthma. The patient population was not further specified as the results from the literature search were anticipated to answer, which patient characteristics would qualify for omalizumab therapy.
   2. Intervention - Omalizumab (dosed according to IgE level and bodyweight according to the Summary of Products Characteristics [SmPC](58)) administered subcutaneously every two or four weeks dependent on dose, on top of standard care.
   3. Comparator - Placebo on in addition to standard care.
   4. Outcome(s) (MCIDs)– of critical importance: exacerbation rate (at least an average reduction in the annual number of exacerbations of 25%, corresponding to a minimum reduction of 0.5 exacerbations per year); OCS, 1) average %-reduction in daily dose [maintenance-treatment] (at least 20% and at least 2.5 mg prednisolone-equivalent dose, 2) percentage of patients discontinuing OCS (at least 5 percentage points), 3) percentage of patients experiencing ≥50% reduction of OCS dose (at least 10 percentage points).
2. Which children (6-18 years) with severe allergic asthma should be offered treatment with omalizumab?
   1. Population - Patients 6 to 18 years of age with severe, allergic asthma. The patient population was not specified in further details, because the results from the literature search was anticipated to answer, which patient characteristics would qualify for omalizumab therapy.
   2. Intervention - Omalizumab (dosed according to IgE level and bodyweight according to the Summary of Products Characteristics [SmPC]) administered subcutaneously every two or four weeks dependent on dose, on top of standard care.
   3. Comparator - Placebo on top of standard care.
   4. Outcome(s) (MCIDs)– of critical importance: exacerbation rate (at least an average reduction in the annual number of exacerbations of 25%, corresponding to a minimum reduction of 0.5 exacerbations per year); OCS, 1) average %-reduction in daily dose [maintenance-treatment] (at least 20% or 2.5 mg prednisolone-equivalent dose), 2) percentage of patients discontinuing OCS (at least 5 percentage points), 3) percentage of patients experiencing ≥50% reduction of OCS dose (at least 10 percentage points).

Table 1: Search strings and results – clinical questions

| **#** | **MEDLINE** | **n** | **#** | **Embase** | **n** | **#** | **CDSR, DARE, CENTRAL** | **n** | **Annotations** |
| --- | --- | --- | --- | --- | --- | --- | --- | --- | --- |
| 1 | ast?ma$.af. | 170,126 | 1 | ast?ma$.af. | 269,016 | #1 | ast*ma* | 26,313 | Records with the term asthma. |
| 2 | (mepolizumab$ or reslizumab$ or omalizumab$).af. | 1,913 | 2 | (mepolizumab$ or reslizumab$ or omalizumab$).af. | 6,556 | #2 | (mepolizumab* or reslizumab* or omalizumab*) | 622 | Records with the generic names mepolizumab, reslizumab or omalizumab. |
| 3 | (nucala$ or cinqa?r$ or xolair$).af. | 135 | 3 | (nucala$ or cinqa?r$ or xolair$).af. | 1,020 | #3 | (nucala* or cinqa*r* or xolair*) | 65 | Records with relevant trade names. |
| 4 | 2 or 3 | 1,917 | 4 | 2 or 3 | 6,558 | #4 | #2 or #3 | 634 | The sum of generic names and trade names. |
| 5 | 1 and 4 | 1,294 | 5 | 1 and 4 | 4,745 | #5 | #1 and #4 | 520 | Intersection of records with the term asthma and generic/trade names. |
| **Filters** | | | | | | | | | |
| 6 | exp Animals/ not Humans/ | 4,327,457 | 6 | exp animal/ not human/ | 4,671,970 |  |  |  | Identification of studies that include animals but not humans. |
| 7 | 5 not 6 | 1,291 | 7 | 5 not 6 | 4,714 |  |  |  | Removal of studies only including animals. |
|  | | | 8 | limit 7 to embase | 2,409 |  |  |  | Limit to records in Embase. |
|  |  |  | 9 | limit 8 to (article or erratum or "review") | 1,067 |  |  |  | Limit to the publication types article, erratum or review. |
| **Systematic reviews** | | | | | | | | | |
| 8 | limit 7 to systematic reviews | **71** | 10 | limit 9 to (meta analysis or "systematic review") | **53** |  | CDSR:  DARE: | **20**  **6** | Limit to systematic reviews and meta-analyses by the use of filters and publication types. The databases CDSR and DARE only contain reviews. |
| **Randomised controlled trials** | | | | | | | | | |
| 9 | (random$ or double-blind$ or placebo$).af. | 1,203,874 | 11 | (random$ or double-blind$ or placebo$).af. | 1,584,234 |  |  |  | Filter to identify randomised controlled trials. |
| 10 | 7 and 9 | **316** | 12 | 9 and 11 | **364** |  | CENTRAL: | **494** | Limit result to randomised controlled trials. The database CENTRAL is pre-filtered to contain only references to randomised trials. |
|  | | | | | | | | | |
| af | "All fields": The search is completed in all available database fields, including title, abstract and thesaurus categories. | | | | | | | | |
| exp | "Explode": More specific thesaurus subcategories are included in addition to the stated category. | | | | | | | | |
| / | Indicates a search in a thesaurus category, e.g. a Medical Subject Heading (MeSH). | | | | | | | | |
| ? | None or one characters. | | | | | | | | |
| $/* | None or more characters. | | | | | | | | |

Table 2 - Study characteristics of the included studies (children)

|  | **Study design** | **Follow-up** | **Age** | **Outcome measure** | **Description of asthma assessment up to study start** | **Asthma severity** | **Refractory asthma (exacerbations, symptoms FEV1…)** | **Lung function < 80 %** | **Inhalations-**  **steroid** | **2nd controller** | **Oral steroid** | **Number of patients (randomised)** | **Notes** |
| --- | --- | --- | --- | --- | --- | --- | --- | --- | --- | --- | --- | --- | --- |
| **Busse 2011**  US | ICATA  randomized, double-blind, placebo-controlled, parallel-group, multicentre trial of omalizumab in 419 inner-city children, adolescents, and young adults | 60 weeks (hereof 12 weeks wash-in, as data in ITT analysis is for 48 weeks) | 6–20 | Exacerbation rate  Lung function  Asthma control  SAE  Drop out  Sick leave | study physicians determined the appropriate asthma regimen on the basis of symptoms, percentage of predicted forced expiratory volume in 1 second (FEV1), and current level of therapy, with the goal to achieve disease control. This regimen was administered for a 4-week run-in period.  + education about relevant environmental allergen remediation | Mild (28-25 %) Severe (53-55 %)  + at least one positive perennial allergen skin test | Patients receiving long-term therapy for disease control were also required to have symptoms of persistent asthma or evidence of uncontrolled disease as indicated by hospitalization or unscheduled urgent care in the 6 to 12 months preceding study entry. Those not receiving long-term control therapy were eligible for enrolment only if they had both persistent symptoms and uncontrolled asthma. | FEV1:FVCx100 v. baseline:  77.0 (9.9) | After run-in: mean daily increase in the budesonide-equivalent dose of an inhaled gluco corticoid of 204 µg (95 % confidence interval [CI], 161 to 247) | After run-in: mean increase in the pro- portion of participants receiving a long-acting beta-agonist (LABA) of 42 % (95 % CI, 37 to 47) | Allowed if exacerbation | 419 |  |
| **Sorkness 2013**  US | Post hoc analysis from Busse 2011 | 60 weeks (48 weeks) | 6–20 | exacerbations rate  Asthma control |  |  |  |  |  |  |  |  | 4 months. After discontinuation  Oma: + 0.84 symptom age  Placebo: no change  Difference in frequency of exacerbations  every 2. weeks: OR 2.54  every 4. weeks:  Or 1.42  Sub groups with better effect |
| **Lanier 2009**  USA, Canada Colombia, Argentina, Brazil, Poland,, South Africa | Those who remained inadequately controlled were then randomized 2:1 to receive omalizumab or placebo for a 52-week treatment period, consisting of a 24-week fixed-steroid phase, and a 28-week adjustable-steroid phase | 24 (stable steroid) + 28 (adjustable steroid) weeks | 6–12 | exacerbations rate  Asthma control  QoL  SAE  Drop out | Patients entered a 1-week screening phase, followed by an 8-week run-in phase to optimize asthma treatment and establish baseline asthma control measurements. ICS and other asthma control medications could be adjusted during the first 4 weeks of the run-in; further dose adjustments were not permitted during the last 4 weeks of the run-in | Moderate -severe (64%)  + at least one positive perennial allergen skin test and/or a positive radioallergosorbent test | history of exacerbations (>2 within 1 year, >3 within 2 years, or >1 severe exacerbation requiring hospitalization within 1 year before study entry). Inadequately controlled  asthma despite receiving at least medium doses of ICS (200mg/d fluticasone propionate via dry powder inhaler or equivalent) | FEV1 % pred. v. baseline: 86 | Daily dose mean: 515.1  Demanded >200 µg day-1 or equivalent (Step 3 or 4 of NHLBI guidelines | with or without other controller medications.  LABA: 87% | -  allowed  I n the study period 92% of the clinical significant exacerbations were treated with OCS | 627 | Effect on exc. ’trend towards consistency independent of baseline FEV1 % pred. and LABA/non-LABA. |
| **Kulus 2010** | Subgroup from Lanier 2009 | 24 (stable steroid) +28 weeks (adjustable steroid) | 6–12 | exacerbation rate  Asthma control  SAE  Drop out | As Lanier 2009 | Severe | As Lanier 2009, despite: Inadequately controlled severe asthma despite receiving high dose ICS (>500µg/day -1 FP or equivalent) and a LABA, with or without other controller medications | FEV1 v. baseline: 82.1 | Daily dose mean: 744.0  Demand >200 µg day-1 or equivalent (Step 3 or 4 of NHLBI guidelines | with or without other controller medications.  LABA: 100% | 2,6 % | 246 |  |
| **Sly 2017**  Australia | double- blind, randomized, controlled 2-year pilot study | 104 weeks (treatment 5 months.) | 6-15 | exacerbation rate  Lung function  Drop out | - | - | history of a severe asthma exacerbation in the previous winter | FEV1 Z score, mean: -1.06 (omalizumab),  -1.17 (placebo) | Dose, mean: 705 (Omalizumab) 1088 (placebo) | - | - | 27 | Underpowered. Should have been 40 in each group |
| **Teach 2015ab**  USA | PROSE  3-arm, randomized, double-blind, double placebo- controlled, multicenter clinical trial. Compare (1) omalizumab with placebo and (2) omalizumab with a boost in ICS | 17 to 21 weeks | 6-17 | exacerbation rate  Lung function  Asthma control  SAE  Drop out | 4- to 9-month run-in phase during which guidelines-based care was delivered to achieve asthma control. | Moderate -severe  + positive perennial allergen skin test | asthma diagnosis or  symptoms for more than 1 year, 1 or more asthma exacerbations (requiring systemic corticosteroids) or hospitalization within the prior 19 months. | FEV1/FVCx100 v. baseline: 77.8 (9.49) | At least 200ug fluticasone  Sub groups with over and under 500 ug fluticasone | By using a predefined EPR3-based treatment algorithm,  clinicians determined each participant’s controller regimen | - | 478 |  |

Table 3 - Baseline characteristics of the omalizumab trials for children included in the analyses

| **Reference** | **n (total)** | **Sex (% female)** | **Ethnicity (% Caucasian)** | **Age** | **Age(SD) / [range]** | **BMI mean (SD)** | **Moderate asthma (%)** | **Severe asthma (%)** | **# exacerbations previous 12 month** | **Serum IgE (mean)** | **Pre-bronchodilator FEV1 (mean/median % pred.)** | **LABA or other concomitant medication, n (%)** | **Daily dose ICS mean** | **OCS, n (%) and optionally daily dose mean** | **Notes to baseline characteristics  *(Skewed distribution. The degree of asthma, generalisability to Danish population. Other notes)*** |
| --- | --- | --- | --- | --- | --- | --- | --- | --- | --- | --- | --- | --- | --- | --- | --- |
| **Busse 2011** | 419 | 42.0 | 3 (other or mixed race. 60 % black, 47 % Hispanic) | 10.9 | SD 3.5 | (weight 20-150 kg) | 19.0 | 54.0 | - | - | Oma: 92.9±18.7 Placebo:92.2±17.6 | Oma: 115 (55) Placebo: 111 (53) | - |  | The baseline characteristics of the two study groups were similar. Only a small percentage Caucasians, almost ¼ of the parents did not complete high school; almost 1/3 of all parents were unemployed, and the household income for ½ of patients was <$15.000 (ca. kr. 100.000) |
| **Sorkness 2013** | 419 | 42.0 | 3 (other or mixed race. 60 % black, 47 % Hispanic) | 10.9 | SD 3.5 | (Weight 20-150 kg) | 19.0 | 54.0 | - | - | Oma: 92.9±18.7 Placebo:92.2±17.6 | Oma: 115 (55) Placebo: 111 (53) | - |  | Post hoc analyse af Busse 2011 – baseline derfor identisk med Busse 2011 |
| **Lanier 2009** | 627  (safety population) | 32.3 | 60,0 | 8.6 | SD 1.7 | - | 35.0 | 64.0 | 2.6 | 469.7 | 86.4 | 87 % | 515.1 | 1.3 % | Patient demographic and baseline clinical characteristics were well balanced between treatment groups |
| **Kulus* 2010** | 246 | 34.5 | 59,6 | 9,0 | SD 1.73 | - | - | 100.0 | 2.8 | 440 | 82.1 | 100 % | 744.0 | 2.6% | Baseline demographic and clinical characteristics were generally well balanced between both treatment groups, although children in the omalizumab group were slightly older |
| **Sly 2017** | 27 | 50 |  | 11.5 | SD 3.0 |  |  |  | 2.25 | 423.7 | FEV1 Z score, mean: -1.06 (omalizumab),  -1.17 (placebo) |  | 897 |  | no important differences between groups. |
| **Teach 2015** | 513 (randomised)  478 MITT | 36.6 |  | 10.2 | SD 2.93 |  |  |  |  | - | 90.1 |  |  |  | Omalizumab vs. a )placebo and b)ICS boost. Data here are all populations combined. |

Table 4 - Study characteristics of the included studies (adults)

|  | **Study design** | **Follow-up** | **Age** | **Outcome measure** | **Description of asthma assessment up to study start** | **Asthma severity** | **Refractory asthma (exacerbations, symptoms FEV1…)** | **Lung function < 80 %** | **Inhalations-**  **steroid** | **2nd controller** | **Oral steroid** | **Number of patients (randomised)** |
| --- | --- | --- | --- | --- | --- | --- | --- | --- | --- | --- | --- | --- |
| **Ayres 2004** | Randomized, open-label, omalizumab vs. BSC | 12 months | 12-75 years | Exacerbation rate  FEV1  Symptom score  SAE  Drop out?  Sick leave? | No description, smoking >10 pack-years not allowed | Moderate-severe | >=2 yearly exacerbations, with at least 1 acute contact to ED or admission | Not mentioned median is 70-72 % | Moderate-/high-dose | Allowed, but not a demand  (77,9 %) | Allowed (21,2 %) | 312 |
| **Bardelas 2012** | Randomized double-blind,  placebo-controlled trial | 24 weeks | >=12 years | FEV1  ACT  SAE  Drop out  Sick leave | Smokers and >10 pack-years not allowed | Moderate-severe | ACT<19 plus extra demand on nightly or day-symptoms or FEV1<80% expected within 4 weeks | No demand | Minimum medium dose | yes | Not allowed | 271 |
| **Bousquet 2011** | randomized, open-label, omalizumab vs. OAT  ICS and OCS could be adjusted ongoing after clinical assessment | 32 weeks | 12-75 years | exacerbation rate  FEV1%  ACQ  SAE  Drop out | 8 weeks run-in and 4 weeks hereafter with optimising asthma treatment>10 pack-years not allowed | Severe | <=1 yearly exacerbation, GINA 2004 step 3 or 4 clinical features? | 40-80 % | High dose >=800 beclomethasone propionate | yes | Allowed | 404 |
| **Buhl 2002a** | RCT extension phase 24 weeks from Soler 2001. The extension was blinded but additional asthma medicine was set free | In total 52 weeks | 12-75 years | Exacerbations  FEV1  SAE | Run-in with ICS adjustment, Smoking not allowed? | Moderate | Symptoms not closer defined | 40-80 % | 420 to 840 µg/day of  beclomethasone dipropionate (BDP) or its equivalent ICS | Not allowed | Not allowed | 483 out of 546 included in the extension phase |
| **Buhl 2002b** | RCT both core-study and extension phase from Soler 2001.QoL data. The extension was blinded but additional asthma medicine was set free | In total 52 weeks | 12-75 years | AQLQ | Run-in with ICS adjustment, Smoking not allowed? | Moderate | Symptoms not closer defined | 40-80 % | 420 to 840 µg/day of  beclomethasone dipropionate (BDP) or its equivalent ICS | Not allowed | Not allowed | 483 out of 546 included in the extension phase |
| **Busse 2001** | Randomized double-blind,  placebo-controlled trial | 16 weeks steroid stabile and 12 weeks reduction | 12-75 years | Exacerbations  Asthma control?  SAE  Drop out | Run-in with ICS adjustment, Smoking not allowed? | moderate | Symptoms not closer defined | 40-80 % | 420 to 840 µg/day of  beclomethasone dipropionate (BDP) or its equivalent ICS | Not allowed | Not allowed | 525 |
| **Busse 2013** | Randomized double-blind,  placebo-controlled trial | 24 weeks | 12-75 years | Exacerbations  FEV1  SAE  Drop out | Run-in, no details | Moderate-severe maybe? | Symptom score | > 80 % normal lung function | Not described,  But mean dose 489-528 type ICS unknown | allowed | Not allowed | 328 |
| **Chanez 2010** | Randomized double-blind,  placebo-controlled trial | 16 weeks | >=18 years | Exacerbations  Asthma control  Sick leave | Smoking >20 pack-years not allowed | severe | >2 exacerbations preceding year or 1 admission/ED contact due to exacerbation plus frequent dag- or night symptoms | <80 % | High dose >1000 ug beclomethasone dipropionate or equivalent | yes | allowed approx. 23 % | 31 |
| **Finn 2003** | Randomized double-blind,  placebo-controlled trial, QoL analyses on Busse 2001 | 52 weeks, 28 core study and 24 weeks extension | 12-75 years | QoL (AQLQ) | Run-in with ICS adjustment, Smoking not allowed? | moderate | Symptoms not closer defined | 40-80 % | 420 to 840 µg/day of  beclomethasone dipropionate (BDP) or its equivalent ICS | Not allowed | Not allowed | 525 |
| **Hanania 2011** | Randomized double-blind,  placebo-controlled trial | 48 weeks | 12-75 years | exacerbations rate  TASS-symptom score  AQLQ  SAE  Drop out | Smoking >10 pack-years not allowed | severe | >1 yearly exacerbations plus extras demand plus extra demand on nightly or day-symptoms documented in two weeks of the run-in phase | 40-80 % | High dose >=1000 fluticasone | yes | allowed | 850 |
| **Holgate 2003** | Randomized double-blind,  placebo-controlled trial | 32 weeks, 16 weeks add-on phase and 16 weeks, ICS reduction phase | 12-75 years | exacerbations rate  FEV1  Symptom score  QoL (AQLQ)  SAE  Drop out | 6-10 weeks run-in with ICS-optimising | Moderate-severe | Not mentioned | Not mentioned mean 62,9-66,0 % | High dose  Fluticasone >=1000 | only LABA allowed (43,3-49,2 %) | Patients with OCS is reported in another manuscript? | 246 |
| **Hoshino 2012** | Randomize, open-label (Japan) | 16 weeks | 20-75 years | FEV1  AQLQ | Optimising ICS in the run-in period | Severe? High-dose ICS? | Night- or day symptoms | Not mentioned median is 65-68 % | >440 fluticasone propionate | yes, LABA | allowed | 30 |
| **Humbert 2005 INNOVATE** | Randomized double-blind,  placebo-controlled trial | 28 weeks | 12-75 years | exacerbations rate  FEV1  Asthma symptom score  AQLQ  Drop out  SAE | Run-in period with adjustment of medicine and inhalation technique. Current smoking or more than 10 pack-years not allowed | Severe | 2 exacerbations within the preceding year or 1 serious exacerbation with acute contact to ED or admission | 40-80 % | High dose >=800 beclomethasone or >400 fluticasone propionate | yes, LABA | Allowed up to 20 mg (22 %) | 419 which were included in efficacy because of changes in protocol s |
| **Lanier 2003** | Extension from Busse 2001, asthma medicine was set free | 24 weeks extension  In total 52 weeks | 12-75 years | Exacerbations  FEV1  SAE  Drop out | Run-in with ICS adjustment, Smoking not allowed? | Moderate | Symptoms not closer defined | 40-80 % | 420 to 840 µg/day of  beclomethasone dipropionate (BDP) or its equivalent ICS | Not allowed | Not allowed | 460 in the extension phase |
| **Li 2016** | Randomized double-blind,  placebo-controlled trial (china) | 24 weeks | 18-75 years | FEV1%  AQLQ  ACQ  SAE  Drop out | Run-in with adjustment of medicine. Current smoking or more than 10 pack-years not allowed | Moderate-severe | >=2 exacerbations plus symptoms GINA | 40-80 % | Moderate- high | yes |  | 616 |
| **Niven 2008** | Randomized, open-label, subgroup analysis on severe asthma from Ayres et al. 2004 ca 50% of patients. | 1 year | 12-75 years | Exacerbations  FEV1%  Asthma symptom score (Wasserfall)  Mini-AQLQ  Sick leave | No description. Smoking >10 pack-years not allowed | Servere | **>=2 yearly exacerbations, with 1 acute contact to the ED or admission** | Not mentioned | High dose >=1000 beclomatesone ekv. | yes, LABA | allowed | 164 |
| **Ohta 2009** | Randomized double-blind,  placebo-controlled trial (Asian) | 16 weeks | 20-75 years | Exacerbations  FEV1  Asthma symptom score  SAE  Drop out |  | Moderate-severe | Symptoms  if exacerbations patients are excluded from the study | Mentioned as one or more of the symptoms | High dose? | yes | Not allowed | 315 |
| **Rubin 2012** | Randomised open label (Brazil) | 20 weeks | >12 years | Exacerbations  AQLQ  FEV1  SAE  Drop out | No details | Severe | Persistent, uncontrolled, no further details | Nothing mentioned | High dose  >500 fluticasone or equivalent | yes | ? | 116 |
| **Siergiejko 2011** | Sub grou0 analysis of Bousquet 2011 (open label) on patients treated with OCS:  OCS-reduction was outcome | 32 weeks | 12-75 years | OCS-reduction | 8 weeks run-in and 4 weeks hereafter with optimising asthma treatment>10 pack-years not allowed | Severe | <=1 yearly exacerbations, GINA 2004 step 3 or 4 clinical features? | 40-80 % | High dose >=800 BDP | yes | yes | 82 |
| **Soler 2001** | Randomized double-blind,  placebo-controlled trial | 16 weeks steroid stable 8 weeks with reduction and 4 weeks stable | 12-76 years | Exacerbations  FEV1  Symptom score  FEV1%  SAE  Drop out | Run-in, no details? Smoking not allowed? | Moderate | Symptom score | 40-80 % | High dose 500-1200 beclomethasone propionate | Perhaps not allowed? Mentioned in Buhl et al | Not allowed | 546 |
| **Vignola 2004** | Randomized double-blind,  placebo-controlled trial | 28 weeks | 12-75 years | Exacerbations  FEV1  Asthma symptom score (Wasserfall)  AQLQ  SAE  Drop out |  | Moderate-severe asthma and rhinitis | >=2 unscheduled medical visits for asthma during the past year  or >= 3 during the past 2 years |  | Medium dose >400 budesonide | allowed | Not allowed | 405 |

Table 5 - Baseline characteristics of the omalizumab trials for adults included in the analyses

| **Reference** | **n (total)** | **Sex (%**  **female)** | **Ethnicity (% Caucasians)** | **age** | **Age (SD) / [range]** | **BMI mean (SD)** | **Mode-rat e asthma (%)** | **Severe asthma (%)** | **# exacerbations previous 12 month** | **Serum IgE (mean)** | **Pre-bronchodi-lator FEV1 (mean/median % pred.)** | **LABA or other concomitant-ant medication, n (%)** | **Daily dose ICS mean** | **OCS, n (%) and optionally daily dose mean** | **Notes to baseline characteristics  *(Skewed distribution. The degree of asthma, generalisability to Danish population. Other notes)*** |
| --- | --- | --- | --- | --- | --- | --- | --- | --- | --- | --- | --- | --- | --- | --- | --- |
| **Ayres 2004** | 312 | 70.5 |  | 38.4 | 12-73 |  |  |  |  | 167 (with median) | 71.4 | 77.9 | 2000.0 |  | No measurement of serum-IgE in the "best standard care alone" group |
| **Barela’s 2012** | 271 | 66.4 | 75.3 | 41.3 | sd 14.8 | weight |  |  |  | 182.0 | 75.5 | 87.1 |  |  |  |
| **Bousquet 2011** | 400 | 64.8 |  | 45.7 | sd 12.87 |  |  | 1.0 | 2.1 (sd 1.26) | 232.7 | 62.4 | 99.8 | 99.8 | 88 %. 13.1 mg/day | Demographic and background characteristics and baseline medication use were comparable between treatment groups |
| **Buhl 2002a** | 483 | 50.5 | 83.1 | 40.5 | 12-76 | - | 77.2 | 22.8 | - | 212.2 | 70.2 |  | 771.7 | - |  |
| **Buhl 2002b** | 546 | 51.0 |  | 40.0 | 12-76 | - | - | 59.5 | - | 214.5 | 70.0 |  | 770.5 | - | the two treatment groups were well balanced in terms of demographics and baseline clinical characteristics |
| **Busse 2001** | 525 | 59.0 | 89.0 | 39.2 | 12-74 | - | - | - | - |  | 68.0 | - | - | - | No significant difference |
| **Busse 2013** | 333 | 69.0 | 70.5 | 37.0 | SD 15 | weight | - | - | 48.8% had 1 asthma exacerbation. and 12.5% had 2 or more exacerbations |  | 85.8 | 79.3 | 508.2 | - | Patient demographics and baseline characteristics were well balanced between the treatment groups' |
| **Chanez 2010** | 31 | 61.3 | - | 47.7 | Sd 47.4 |  |  |  | 4.4 (sd 2.15) | 220.2 | 63.2 | 100 | 3556 | 22.6 % |  |
| **Finn 2003** | 525 | 59.0 |  | 39.2 | 12-74 |  |  | 99.5 |  | 179.4 | 68.0 |  | 569.0 |  |  |
| **Hanania 2011** | 850 | 65.7 | 74.4 | 44.5 | sd 14.1 | 31.8 (sd 7.6) |  |  | 1.95 (sd 1.85) | 176.9 | 64.9 | 83.0 |  | 17 % | Baseline demographic and clinical characteristics were well-balanced between the 2 treatment groups; however. there were slightly more women in the placebo group (70 % vs. 61 %) |
| **Holgate 2004** | 246 | 60.9 |  | 40.8 | 12-75 |  |  | 100.0 |  | 266.3 | 64.5 | 46.3 | 1369.0 |  | The two treatment groups were comparable at baseline in terms of patient demographic and clinical characteristics |
| **Hoshino 2012** | 30 | 76.7 |  | 55.2 | 20-75 |  |  | 100.0 |  | 265.0 | 66.9 | 100.0 | 827.0 | 30 % | There were no significant differences between the groups in any of the studied parameters |
| **Humbert 2005** | 419 | 66.6 | 78.0 | 43.3 | 12-79 | weight |  | 97.0 |  | 193.6 | 61.3 | 100.0 | 2330.0 |  | With the exception of prior exacerbation history. the demographics and background characteristics of the PITT population were similar |
| **Lanier 2003** | 460 | 58.3 | - | 39.1 | 12-74 | - | - | 100% | - | 179.8 | 68.5 | - | 558.5 | - | Overall. baseline characteristics were comparable for the two treatment groups |
| **Li 2016** | 609 | 53.9 | 0.0 | 46.5 | SD 11.9 |  |  |  | 2.2 (SD0.8) | 275.4 | 63.3 |  |  |  | Both treatment arms were well-balanced in demographic and baseline characteristics |
| **Niven 2008** | 164 | 73% |  | 39.0 | 12-73 |  |  | 100.0 |  |  | 64.9 | 98.7 |  | 99.0 |  |
| **Ohta 2009** | 315 | 55% | 0.0 | 49.0 | 20-75 | 61.9 | 4% | 96% |  | 254.1 | 73.4 | 51% | 1169.0 | 10 % | There was no significant imbalance between the groups in demographic and background characterise- tics (Table |
| **Rubin 2012** | 116 | 77% | 66% | 44.5 | 13-72 |  |  |  |  | 234.0 |  |  |  |  | The two groups were comparable in all aspects including baseline IgE levels. There was no significant difference between groups with respect to demographic data |
| **Siergiejko 2011** | 82 | 66% | 100.0 | 45.4 | 12-75 | 74.4 |  | 100.0 | 3 (SD1.7) | 240.8 | 61.0 |  |  | 100 %. doses: 13 mg/day | Patient demographic and background characteristics were similar between treatment groups |
| **Soler 2001** | 546 | 50.9 | 91.2 | 39.5 | 12-76 | - | 78.2 | 21.8 | - | 214.4 | 69.9 |  | 770.6 | - | The treatment groups were well balanced with respect to demographics |
| **Vignola 2004** | 405 | 55% |  | 38.4 | SD 14.73 |  |  | 90.0 | 2.1 |  | 78.2 | 39 % | 871.6 |  | Demographic characteristics were similar in both groups |

Table 6 - Omalizumab to adults with severe, allergic asthma. The overall quality of evidence is estimated as very low. The evidence quality for sick leave could not be performed due to lack of data.

| **Certainty assessment** | | | | | | | **№ of patients** | | **Effect** | | **Certainty** | **Importance** |
| --- | --- | --- | --- | --- | --- | --- | --- | --- | --- | --- | --- | --- |
| **№ of studies** | **Study design** | **Risk of bias** | **Inconsistency** | **Indirectness** | **Imprecision** | **Other considerations** | **Omalizumab** | **Placebo** | **Relative (95 % CI)** | **Absolute (95 % CI)** |  |  |
| Exacerbation rate | | | | | | | | | | | | |
| 5 | randomised trials | serious ^a^ | not serious ^b^ | serious ^c^ | serious ^d^ | none | -/1023 | -/900 | **Rate ratio 0.63** (0.50 to 0.79) | 0.74 (0.42 to 1.0) | ⨁◯◯◯ VERY LOW | CRITICAL |
| Number of patients who experience 0 exacerbations annually | | | | | | | | | | | | |
| 11 | randomised trials | serious ^e^ | not serious | serious ^c^ | not serious | none | 1703/2152  (79.1 %) | 1464/2018  (72.5 %) | **RR 1.11** (1.07 to 1.14) | **82 more per 1.000** (from 52 more to 104 more) | ⨁⨁◯◯ LOW | CRITICAL |
| Percentage of patients who experience ≥50% reduction of oral corticosteroid treatment* | | | | | | | | | | | | |
| 1 | randomised trials | serious ^f^ | serious ^g^ | not serious | serious ^h^ | none | In the omalizumab the OCS usage was reduced by 45 % (SD 50.22), while the OCS usage in the placebo group was increased by 18.3 % (SD 85.13). p = 0.013 | | | | ⨁◯◯◯ VERY LOW | CRITICAL |
| Percentage of patients who are discontinued oral corticosteroid-maintenance treatment | | | | | | | | | | | | |
| 1 | randomised trials | serious ^f^ | serious ^g^ | not serious | very serious ^i^ | none | 19/59 (32.2 %) | 3/23 (13.0 %) | **RR 2.47** (0.81 to 7.55) | **192 more per 1.000** (from 25 fewer to 854 more) | ⨁◯◯◯ VERY LOW | CRITICAL |
| Lung function FEV_1_ | | | | | | | | | | | | |
| 5 | randomised trials | serious ^e^ | not serious | serious ^c^ | not serious | none | An effect on FEV1 was observed in the omalizumab group, but not achieving clinical relevance. | | | | ⨁⨁◯◯ LOW | IMPORTANT |
| Asthma control (ACQ5. ACT, Wasserfallen asthma symptom score)) | | | | | | | | | | | | |
| 5 | randomised trials | serious ^e^ | serious ^j^ | serious ^c^ | serious ^d^ | none | 1220 | 1067 | - | SMD **0.36 lower** (0.58 lower to 0.13 lower) | ⨁◯◯◯ VERY LOW | IMPORTANT |
| QoL (AQLQ and SGRQ) | | | | | | | | | | | | |
| 4 | randomised trials | serious ^e^ | very serious ^k^ | serious ^c^ | serious ^l^ | none | 816 | 705 | - | MD **0.58 higher** (0.04 higher to 1.12 higher) | ⨁◯◯◯ VERY LOW | IMPORTANT |
| Serious adverse events (SAEs) | | | | | | | | | | | | |
| 13 | randomised trials | serious ^e^ | very serious ^m^ | serious ^c^ | not serious | none | 153/2691 (5.7 %) | 144/2354 (6.1 %) | **RR 0.86** (0.68 to 1.07) | **9 fewer per 1.000** (from 4 more to 20 fewer) | ⨁⨁◯◯ LOW | IMPORTANT |
| Drop-out | | | | | | | | | | | | |
| 11 | randomised trials | not serious | serious ^o^ | serious ^c^ | not serious | none | 256/2287 (11.2 %) | 330/2270 (14.5 %) | **RR 0.77** (0.59 to 1.01) | **33 fewer per 1.000** (from 1 more to 60 fewer) | ⨁⨁◯◯ LOW | IMPORTANT |
| Sick-leave - not reported | | | | | | | | | | | | |
| - | - | - | - | - | - | - |  | | | | - | IMPORTANT |

**CI:** Confidence interval; **RR:** Risk ratio; **MD:** Mean difference; **SMD:** Standardised mean difference

a. Two of the studies were open label, hence a significant risk of bias. Therefore, degraded for risk of bias.

b. A certain inconsistency in the estimates, but could be explained by the two studies showing the most pronounced effect were open label. The population in the study producing the most pronounced heterogeneity, were all patients with severe asthma, which could explain the pronounced effect. Therefore, no degradation of inconsistency

c. A lack of systematic diagnosing of severe asthma is observed in most of the studies, and the study populations included moderate asthma. This does not apply to Danish conditions, and the clinical question asked. Therefore, a degradation for indirectness.

d. 95 % CI overlaps the minimal important clinically difference, thus a degradation of imprecision.

e. A majority of studies are labelled ”unclear” in the assessment of risk of bias, both in terms of the randomisation procedure as well as blinding. Furthermore, unblinded studies are included, thus a degradation of risk of bias.

f. The study is open label and both randomization procedure as well as blinding is unclear. Thus, a degradation of risk of bias.

g. Only a single study with relatively few included patients is the basis of this outcome measure. A degradation of inconsistency because results from a single study can not assess whether data from other studies will show results that are alike.

h. Only very few patients were included in the analysis, and no estimate of uncertainty was presented. Therefore, a degradation of imprecision.

i. Only very few patients were included in the analysis, 95 % CI was wide and included estimates that could lead to different clinical decisions. Therefore, a degradation of imprecision.

j. Heterogeneity was concluded by visual inspection. One study added heterogeneity and different questionnaires were used in the different studies. Therefore, a degradation in inconsistency.

k. Heterogeneity was concluded by visual inspection. One study added heterogeneity which could not be explained Therefore, a degradation of inconsistency.

l. 95 % CI was wide and included estimates that could lead to different clinical decisions. Therefore, a degradation of imprecision.

m. Heterogeneity was concluded by visual inspection. One study added heterogeneity and different questionnaires were used in the different studies. Therefore, a degradation in inconsistency.

n. Relatively few patients were included in the analyses and the event rate was low in two of the studies. Therefore, a degradation of imprecision.

o. Heterogeneity was concluded by visual inspection. One study added heterogeneity which could not be explained Therefore, a degradation of inconsistency.

Table 7 - Omalizumab to children with severe, allergic asthma. The overall quality of evidence is estimated as very low.

| **Certainty assessment** | | | | | | | **№ of patients** | | **Effect** | | **Certainty** | **Importance** |
| --- | --- | --- | --- | --- | --- | --- | --- | --- | --- | --- | --- | --- |
| **№ of studies** | **Study design** | **Risk of bias** | **Inconsistency** | **Indirectness** | **Imprecision** | **Other considerations** | **Omalizumab** | **Placebo** | **Relative (95 % CI)** | **Absolute (95 % CI)** |  |  |
| **Exacerbation rate** (follow up: mean 24 weeks) | | | | | | | | | | | | |
| 1 | randomised trials | not serious | serious ^a^ | serious ^b^ | very serious ^c^ | none | -/166 | -/80 | **Rate ratio 0.66** (0.44 to 0.99) | 1.32 (0.88 to 1.98) | ⨁◯◯◯ VERY LOW | CRITICAL |
| Number of patients who experience 0 exacerbations annually (follow up: mean 48 weeks) | | | | | | | | | | | | |
| 1 | randomised trials | not serious | serious ^a^ | very serious ^b.d^ | serious ^e^ | none | 145/208  (69.7 %) | 108/211  (51.2 %) | **RR 1.36** (1.16 to 1.60) | **184 more per 1.000** (from 82 more to 307 more) | ⨁◯◯◯ VERY LOW | CRITICAL |
| Asthma control (follow up: mean 17-48 weeks; assessed with: ACT/C-ACT. MCID ACT = 3 point; Scale from: 5/0 to 25/27) | | | | | | | | | | | | |
| 2 | randomised trials | not serious | not serious ^f^ | very serious ^b.g^ | serious ^e^ | none | 164 | 164 | - | MD **1.09 higher** (0.89 higher to 1.28 higher) | ⨁◯◯◯ VERY LOW | IMPORTANT |
| Serious adverse events (follow up: mean 17-48 weeks) | | | | | | | | | | | | |
| 4 | randomised trials | not serious | not serious | serious ^b^ | serious ^h^ | none | 19/652 (2.9 %) | 39/388 (10.1 %) | **RR 0.40** (0.24 to 0.67) | 60 fewer per 1.000 (from 33 fewer to 76 fewer) | ⨁⨁◯◯ LOW | IMPORTANT |
| Dropout (follow up: mean 17-48 weeks) | | | | | | | | | | | | |
| 5 | randomised trials | not serious | not serious | serious ^b^ | not serious | none | 60/659 (9.1 %) | 43/397 (10.8 %) | **RR 0.83** (0.56 to 1.22) | 18 fewer per 1,000 (from 24 more to 48 fewer) | ⨁⨁⨁◯ MODERATE | IMPORTANT |
| Sick leave(follow up: mean 17-48 weeks) | | | | | | | | | | | | |
| 3 | randomised trials | not serious | not serious ^j^ | serious ^b^ | not serious | none | 467 | 300 | - | MD **0.53 lower** (1.4 lower to 0.33 higher) | ⨁⨁⨁◯ MODERATE | IMPORTANT |
| Lung function: Percentage who experience an improvement of at least 12 % (follow up: mean 17-48 weeks) | | | | | | | | | | | | |
| 3 | randomised trials | not serious | not serious | serious ^b^ | serious ^e^ | none | No significant difference between omalizumab and placebo on measured FEV1% expected and/or FEV1:FVC ratio | | | | ⨁⨁◯◯ LOW | IMPORTANT |
| Quality of life (follow up: mean 24 weeks; assessed with: PAQLQ) | | | | | | | | | | | | |
| 1 | randomised trials | not serious | serious ^a^ | serious ^b^ | serious ^e^ | none | No significant difference (Least squares mean difference 0.04 in favour of omalizumab (p = 0,676)) | | | | ⨁◯◯◯ VERY LOW | IMPORTANT |
| Reduction in ICS | | | | | | | | | | | | |
| 2 | randomised trials | not serious | serious ^k^ | Serious ^b^ | serious ^e^ | none | Two studies assessed this. In Lanier et al, which is the study closest to the Danish conditions, no clinical relevant effect was observed. | | | | ⨁◯◯◯ VERY LOW | CRITICAL |

**CI:** Confidence interval; **RR:** Risk ratio; **MD:** Mean difference

a. Only a single study with relatively few included patients is the basis of this outcome measure. A degradation of inconsistency because results from a single study can not assess whether data from other studies will show results that are alike.

b. A lack of systematic diagnosing of severe asthma is observed in most of the studies, and the study populations included moderate asthma. This does not apply to Danish conditions, and the clinical question asked. Therefore, a degradation for indirectness.

c. Only a single study with relatively few included patients is the basis of this outcome measure. The data are from a subgroup of the main study and due to a lack of sufficient sample size, the study can not show the outcome with the required statistical significance. Therefore, a degradation for imprecision.

d. The study included ’inner city’-children, where the percentage of Caucasians was < 3 %, and the socioeconomic status was different compared to Danish numbers. The Expert Committee judged that this would lead to a degradation of indirectness.

e. The analysis of this effect measure was based on relatively few individuals. Therefore, a degradation for imprecision.

f. There are very little overlap between the confidence intervals, but the inconsistency can be explained by one of the studies being a subgroup analysis of children with severe asthma. Therefore, no degradation of inconsistency.

g. The study included ’inner city’-children, where the percentage of Caucasians was < 3 %, and the socioeconomic status was different compared to Danish numbers. The Expert Committee judged that this would lead to a degradation of indirectness.

h. There are relatively few individuals in the analysis and the event rate is low in to of the studies. Therefore, a degradation for imprecision.

i. 95 % confidence intervals are wide and contain estimates which would lead to different clinical decisions. Therefore, a degradation of imprecision.

j. There are very little overlap between the confidence intervals, but the inconsistency can be explained by one of the studies being a subgroup analysis of children with severe asthma. Therefore, no degradation of inconsistency.

k. Two of the studies present this with results which are not alike. Therefore, a degradation of inconsistency.

# Narrative review of studies on lung function among omalizumab users compared to standard of care or placebo

Adults

Average change in lung function measured by FEV1:

Three studies presented complete data, and of these two where the effect was statistically significant, but none of the studies showed clinical relevance

**Bardelas** et al 2012 showed that omalizumab was associated with a reduction of FEV1 of -80.00ml (95% CI -190.00;30.00) over a period of 24 weeks

**Busse** et al 2013 showed that omalizumab increased FEV1 by 81 ml (95% CI 10;152) over a period of 24 weeks

**Bousquet** et al 2011 showed that omalizumab increased FEV1 by 130 ml (95% CI 30;230) over a period of 32 weeks

Remaining studies did not present complete data for the comparative analysis, whereof only one study (Niven et al 200) showed a statistical significant effect, which also surpassed the MCID.

**Vignola** et al 2004 showed that omalizumab increased FEV1 by 73 ml compared to placebo. No 95% CIs were presented

**Hoshino** et al 2012 showed that omalizumab increased FEV1 by around 200ml compared to an increase around 20 ml in the placebo-arm of the study.

**Humbert** et al 2005 showed that omalizumab increased FEV1 by 190 ml compared an increase of 96 ml in the placebo-arm

**Ohta** et al 2009 showed that omalizumab increased FEV1 by 39 ml compared to a decrease of FEV1 by 24 ml in the placebo-arm

**Rubin** et al 2012 showed that omalizumab increased FEV1 by 130 ml compared to a decrease of 3 ml in the placebo-arm (p=0.049)

**Niven** et al 2008 (a subgroup-analysis, severe asthma, from Ayres et al) showed that omalizumab increased FEV1 by 320 ml (p<0.05) compared to control-group.

**Lanier** et al 2003 (extension-phase of Soler et al 200) showed a difference in the lung function after extension phase of 52 ml in favor of the omalizumab-group (p=0.16).

Because the majority of the studies showed a difference less than the predefined MCID of 200 ml the Expert Committee considered that omalizumab’s effect on lung function measured by FEV1 was present but not achieving the predefined clinical relevance.

FEV1% of expected

Studies presenting this outcome have insufficiently presented the data in terms of including them into a meta-analysis

**Bousquet** et al 2011 showed that omalizumab increased the FEV1% by 4.4% (1.2-7.6) versus placebo

**Busse** et al 2001 found that in the stable steroid-phase of the study omalizumab showed an increase of 4.3% compared to an increase of 1.4% among controls

**Soler** et al 2001 found that in the stable steroid-phase of the study omalizumab showed an approximate increase of 2-3% (read on a graph) versus placebo.

**Niven** et al 2008 showed a statistical significant increase of approximately 11% in favor of omalizumab compared to placebo

**Li** et al 2016 showed a statistical significant increase of 4.1% (p=0.001) in favor of omalizumab compared to placebo

These data did not change the Expert Committee’s assessment of the effect of on lung function was above the predefined limit for clinical relevance.

Results adults asthma control

A series of studies did not present a sufficient estimate to be included in the meta-analysis. None of the studies presented ACQ or ACT. Out of these studies, a statistical significant effect was observed in asthma control/-symptoms in five studies and no effect was presented in three studies

Statistical significant differences between omalizumab treatment arm and control arm was presented in the following studies:

- Busse 2001: Asthma-symptom score showed a statistically significant improvement in the stabile phase and in the reduction phase (presented by graph/figure with p-values)

- Soler 2001: Total asthma-symptom score showed statistically significant improvements among omalizumab treatment arm versus placebo arm. A statistical significant difference was shown at some times during both the steroid stabile phase and the reduction phase (presented by graph/figure).

- Humbert 2005: Presented an improvement in the omalizumab treatment arm versus placebo (p=0.039)

- Ayres 2004: Showed an improvement of the Wasserfall symptom score in favor of the omalizumab treatment group versus placebo, of 6.2 and 0.7 (p<0.001), respectively.

- Niven 2008, a subgroup analysis of Ayres of patients with severe asthma showed an improvement in the omalizumab group compared to placebo (p<0.001).

No differences were observed in the following studies:

- Busse 2013: described night- and day-symptom score separately and no differences between the omalizumab and placebo were shown

- Chanez 2010: No statistical significant differences in the different ways of presenting day- and night-symptoms.

- Ohta: No statistically significant differences in asthma-symptom store.

The Expert Committee concluded, that the results of these studies were in line with the meta-analysis results.

**Figure 1:** Mean difference quality of life among adults


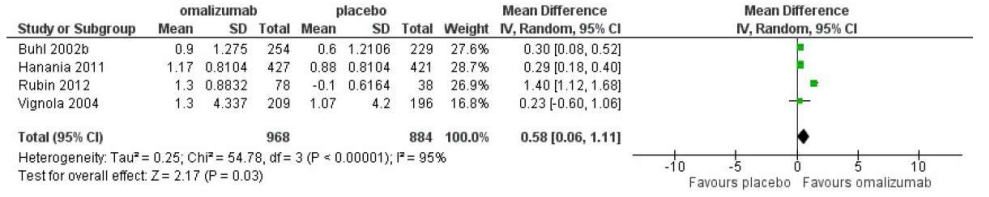


**Figure 2:** Risk ratio for drop out in the studies

2a adults:


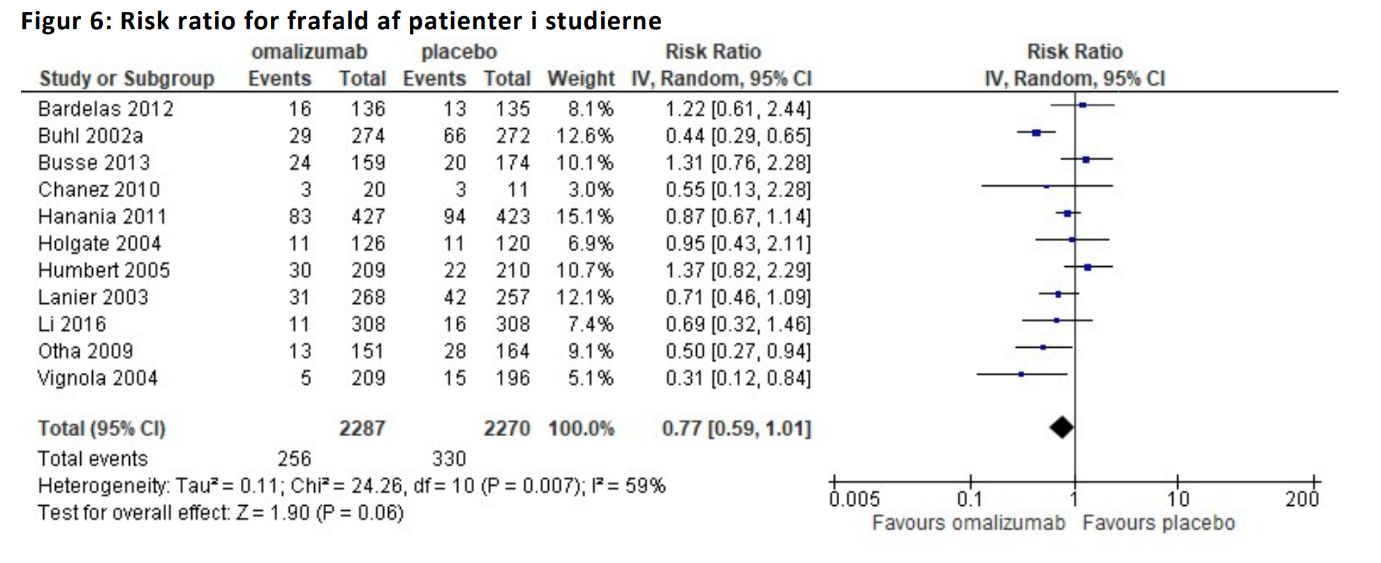


2b children:

**
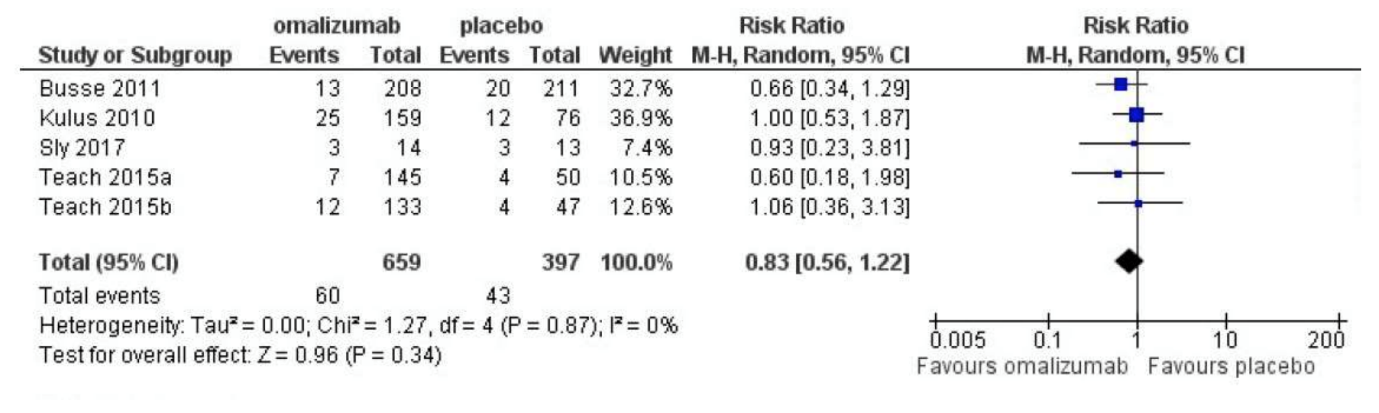
**

**Figure 3:** Risk ratio for serious adverse events

3a adults:
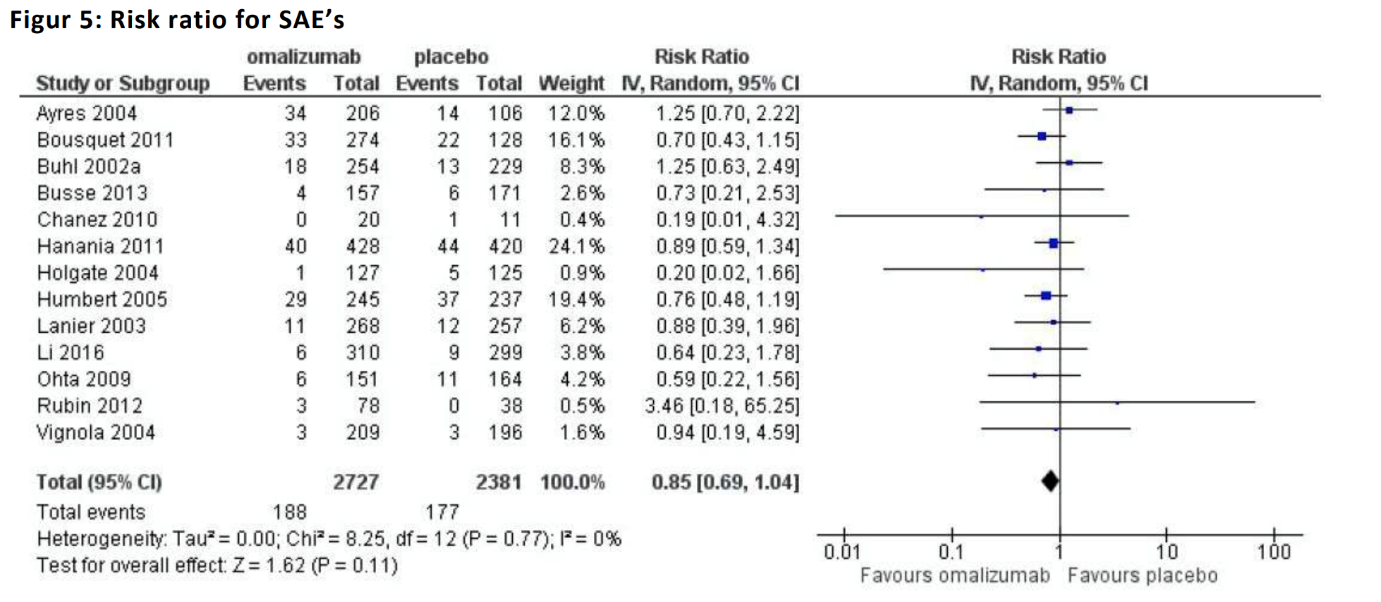


3b Children


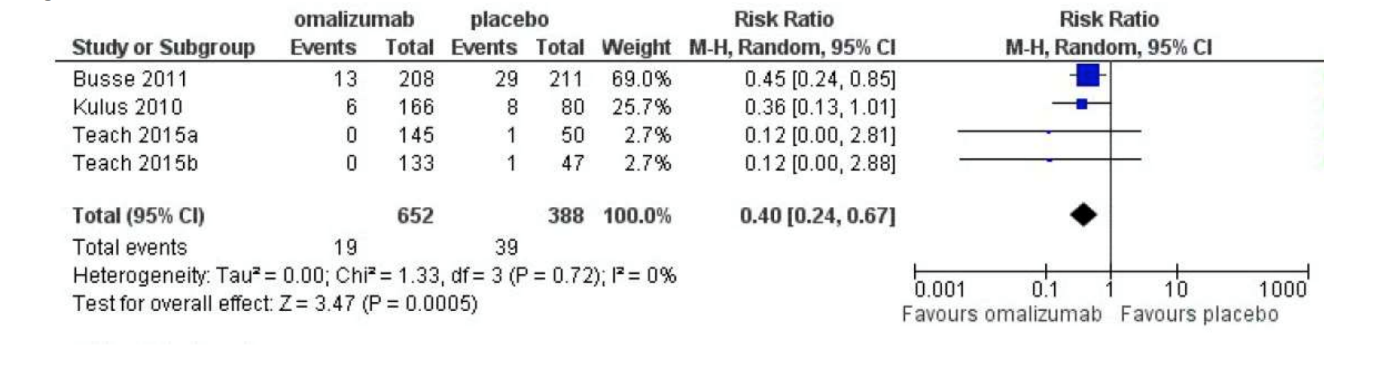

Supplement: Supplementary file 1 — Additional file 1. Appendix. [file 13223_2020_442_MOESM1_ESM.docx]
